# In Vivo Magnetic Resonance Thermometry for Brain and Body Temperature Variations in Canines under General Anesthesia

**DOI:** 10.3390/s22114034

**Published:** 2022-05-26

**Authors:** Keonil Kim, Jisoo Ahn, Kwangyong Yoon, Minjung Ko, Jiyoung Ahn, Hyesung Kim, Jihyeon Park, Chulhyun Lee, Dongwoo Chang, Sukhoon Oh

**Affiliations:** 1 Bio-Chemical Analysis Team, Center for Research Equipment, Korea Basic Science Institute, Cheongju 28119, Korea; kki2021@edevicesolution.com (K.K.); chulhyun@kbsi.re.kr (C.L.); 2 Department of Bio-Analytical Science, University of Science and Technology, Daejeon 34113, Korea; 3 Section of Veterinary Medical Imaging, College of Veterinary Medicine, Chungbuk National University, Cheongju 28644, Korea; dhm01191@hanmail.net (J.A.); apollo02@naver.com (K.Y.); musuri0918@naver.com (M.K.); mellowout79@gmail.com (J.A.); star31109@gmail.com (H.K.); tgu02093@naver.com (J.P.)

**Keywords:** MR thermometry, proton resonance frequency shift, general anesthesia, brain temperature, 7T MRI

## Abstract

The core body temperature tends to decrease under general anesthesia. Consequently, monitoring the core body temperature during procedures involving general anesthesia is essential to ensure patient safety. In veterinary medicine, rectal temperature is used as an indicator of the core body temperature, owing to the accuracy and convenience of this approach. Some previous studies involving craniotomy reported differences between the brain and core temperatures under general anesthesia. However, noninvasive imaging techniques are required to ascertain this because invasive brain temperature measurements can cause unintended temperature changes by inserting the temperature sensors into the brain or by performing the surgical operations. In this study, we employed in vivo magnetic resonance thermometry to observe the brain temperatures of patients under general anesthesia using the proton resonance frequency shift method. The rectal temperature was also recorded using a fiber optic thermometer during the MR thermometry to compare with the brain temperature changes. When the rectal temperature decreased by 1.4 ± 0.5 °C (mean ± standard deviation), the brain temperature (white matter) decreased by 4.8 ± 0.5 °C. Furthermore, a difference in the temperature reduction of the different types of brain tissue was observed; the reduction in the temperature of white matter exceeded that of gray matter mainly due to the distribution of blood vessels in the gray matter. We also analyzed and interpreted the core temperature changes with the body conditioning scores of subjects to see how the body weight affected the temperature changes.

## 1. Introduction

General anesthesia is known to reduce the body temperature of patients; this reduction in temperature can result in hypothermia, which downregulates the immune and platelet functions, thereby delaying patient recovery and causing cardiac complications [1]. Hypothermia can also decrease the neuronal activity so that the level of oxygen delivery or consumption in the brain can be controlled [2,3]. General anesthesia affects the thermoregulation mechanism of the body against hypothermic symptoms such as vasoconstriction and shivering. Consequently, thermoregulation does not function normally [4]; the body temperature can be further reduced by the temperature of the operating room, ventrotomy, or by metabolic downregulation. In patients under general anesthesia, this reduction in body temperature is monitored by measuring the core body temperature, which can be measured at various anatomical locations such as the pulmonary artery, tympanum, esophagus, bladder, and rectum, depending on the type of surgery and the condition of the patient. However, these locations each have their own advantages and disadvantages. For instance, measuring temperature at the pulmonary artery is highly accurate; however, it is also an invasive method. In contrast, measuring temperature at the tympanum is simple and noninvasive but offers a relatively low accuracy. In veterinary medicine, the temperatures at the rectum and esophagus are considered to be the core body temperature. However, inserting a temperature sensor into the esophagus is difficult and risks scarring the esophagus of the subject. Thus, rectal temperature is generally used to monitor the core body temperature.

Previous studies aiming to differentiate between core body temperatures obtained their data using invasive methods [5,6]. By directly inserting a temperature sensor into the brain through a craniotomy, a temperature difference of approximately 1.5–4.7 °C was observed between the brain and core body (measured rectally). This variation between the core body and brain temperatures highlights the necessity of accurate brain temperature measurements. Nevertheless, the abovementioned approach of inserting a temperature sensor via a craniotomy suffers from three limitations. First, it is an invasive technique that requires trained professionals owing to the risk of infection and bleeding. Furthermore, the invasive condition could increase the uncertainty in the brain temperature changes and thus that of the temperature difference. The reduction in temperature due to the craniotomy and the increase due to inflammation caused by the insertion of the sensor make the results inaccurate [7]. Second, this approach does not indicate the temperature gradient associated with the reduction in core body temperature under general anesthesia. Erickson et al. [6] monitored the gradient between the brain and core body temperatures under general anesthesia through invasive procedures and maintained the normal body temperature using a heating pad to compensate for the temperature loss during the craniotomy. This artificial temperature correction can reduce the accuracy and reliability of the results mainly because of the uncertain amount of temperature change due to the heating pad. Third, this approach only measures the temperature values at the locations of the temperature sensors; monitoring changes in temperature throughout the brain tissue is difficult. Considering these issues, it is essential to explore and develop noninvasive methods for monitoring temperature changes throughout the brain.

Noninvasive methods of monitoring brain temperature variations using magnetic resonance imaging (MRI) have been proposed; these methods are known as MRI-based thermometry techniques. Among these, the proton resonance frequency shift (PRFS) method is used extensively [8,9]. This method converts the phase difference at the time interval between MRI phase scans to a temperature difference. The PRFS method is more sensitive to changes in temperature than other MR thermometry techniques, for example T_1_, T_2_, or diffusion coefficient variations caused by temperature changes [8,9].

In this study, we acquired the MR temperature images to measure the variations in the brain temperature of canines under general anesthesia using the noninvasive PRFS method in accumulative form with multiple phase images obtained at 7 T MRI. The rectal temperature, as a core body temperature, was also recorded using the fiber optic thermometer during the MR thermometry to compare with the brain temperature changes. Furthermore, we analyzed the mechanism of the reduction in brain temperature under general anesthesia, based on the distribution of blood vessels at gray/white matters. The body conditioning score (BCS) of the subjects was also interpreted to analyze the relationship between subject’s weight and the core temperature changes.

## 2. Methods

This study was approved by the Institutional Animal Care and Use Committee, Chungbuk National University (CBNUA-1306-19-01). The methods employed in the experiments are detailed in the following subsections.

The conventional PRFS method measures the phase changes in two MR phase images according to the temperature changes during specific time intervals, then converting these phase changes into temperature changes, as expressed in:

(1) $$\Delta T_{n}=\frac{\Delta \phi_{n}-\Delta \phi_{n.drift}}{\gamma \alpha B_{0}TE}\;$$

where ΔT is temperature changes during specific time intervals, $\Delta \phi_{n}$ is the phase difference between the $n^{\mathrm{th}}$ and ${\left(n+1\right)}^{\mathrm{th}}$ phases, $\Delta \phi_{n.drift}$ is the phase change due to the MRI system phase drift, γ is the gyromagnetic ratio of protons (42.57 MHz/Tesla), *α* is the PRFS coefficient (−0.01 ppm/°C, [10]), *B*_0_ is the main magnetic field strength, and *TE* is the echo time of each MR phase scan.

**Figure 1:**
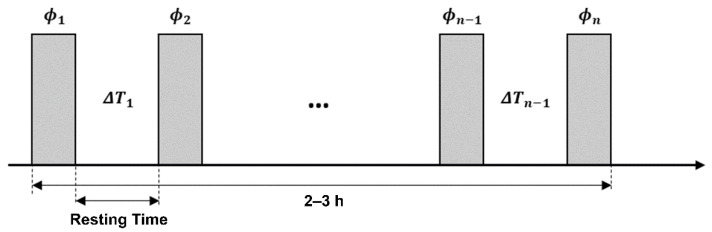
MR temperature experimental scheme. The temperature image ($\Delta T_{n}$) is derived from two successive phase scans ($\phi_{n-1}-\phi_{n}$), whereas the accumulative temperature change (first phase scan to each $\Delta T_{acc.n}$ scan) utilizes the first and the $n^{\mathrm{th}}$ phase scans ($\phi_{1}-\phi_{n}$ ). A resting time of approximately 3 min was adopted between phase scans.

The temperature changes of several consecutive phase images can be sequentially observed in an accumulative form. Thus, we implemented the PRFS method based on the phase images acquired at multiple points in time throughout the period of general anesthesia (Figure 1). A resting time of approximately 3 min between phase scans was adopted to observe temperature changes between appropriate time intervals. The actual phase scan time was done in 43 s, but the resting time needs to be placed between two phase scans to minimize stress of the gradient coil which possibly causes unwanted phase drift. Equation (1) expresses the conventional PRFS, whereas the following equation expresses the accumulative form of PRFS (PRFS_acc_, $\Delta T_{acc}$), which represents the conventional PRFS in the time-integral form:

(2) $$\Delta T_{acc.n}=\Delta T_{1}+\Delta T_{2}+\Delta T_{3}+\ldots +\Delta T_{n-2}+\Delta T_{n-1}\;=\overset{n-1}{\underset{i=1}{\sum }}\left(\frac{\Delta \phi_{i}-\Delta \phi_{i.drift}}{\gamma \alpha B_{0}TE}\right)$$

where $\Delta T_{acc.n}$ indicates the accumulated temperature change from the first to the $n^{\mathrm{th}}$ measurement point, which is converted from the first to the $n^{\mathrm{th}}$ phase change.

Since the phase drift of an MRI system can be influenced by factors other than the temperature change, soy oil phantoms were utilized to measure the unwanted phase drift, as reported by Oh et al. [10]. The phase drift map must be correctly measured to accurately convert the phase change into a temperature image by subtracting the phase drift from the original phase change within the subject. The temporally and spatially varying phase drift $(\Delta \phi_{drift}$) during MR phase scans has various causes, including the heating of passive shimming elements, the alteration of the electrical conductivity of a sample, or time varying magnetic susceptibility, which are difficult to control experimentally [11,12,13]. The phase drift mapping and its correction were applied to the phantom and in vivo cases in this study.

### 2.1. Phantom Study

**Figure 2:**
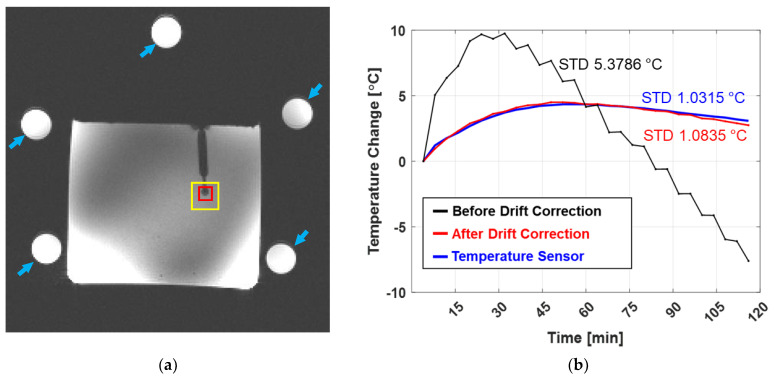

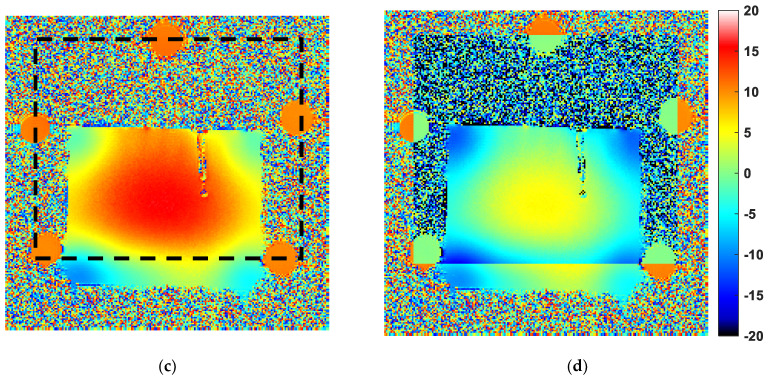
Agar-gel phantom experiment for validation of accumulative PRFS. (**a**) Magnitude MR image of agar-gel phantom. Blue arrows indicate the five reference oil phantoms for phase drift correction. The mean temperature changes (yellow box) were calculated around the tip of the fiber optic temperature sensor, except in the region of the susceptibility artifact (red box). (**b**) Temperature changes measured by fiber optical temperature sensor (red line) and accumulative PRFS techniques (black and blue lines represent changes before and after correction, respectively). Standard deviation (STD) of each temperature measurement was placed with corresponding color. (**c**) PRFS image before phase drift correction. The black dashed box indicates the region of phase drift correction using the five reference phantoms. (**d**) PRFS image after phase drift correction. The temperature in the central area was higher than that at the edges.

To validate the accuracy of the temperature changes measured using the accumulative PRFS technique, agar-gel phantom (agar: 7 g/L, CuSO_4_: 1 g/L, and NaCl: 10 g/L) pre-heated to 25 °C was employed. A fiber optic temperature sensor (1.4 mm diameter, OPT-M, OpSens, Québec, CA, Canada) was inserted into the agar-gel phantom during 2 h of MR temperature scans (30 phase scans with 3 min resting time between each phase scan), so that the temperature sensor measurements were directly comparable to the accumulative MR temperature. MR temperature scans of the agar-gel phantom were acquired using a 7.0 T MRI system (Achieva, Philips, Best, NL, The Netherlands) with a 28-channel receive-array knee coil (Q7000161, QED, Mayfield, KY, USA). The detailed parameters for acquiring the MR temperature images is as follows: gradient echo, field-of-view (FOV): 158 × 158 mm^2^, pixel size: 0.75 × 0.75 mm^2^, slice thickness: 3 mm, number of slices: 7, TR/TE: 100/10 ms, number of averages: 2, flip angle: 15°, bandwidth: 503 Hz/pixel, and acquisition time: 43 s. Five cylindrical oil phantoms (diameter: 17 mm length: 120 mm) were placed around the agar-gel phantom to measure the phase drift. The center-slice of the MR image including oil phantoms, phase drift correction, and the accumulative temperature changes are summarized in Figure 2. No scanner adjustment was applied at each resting time.

The columnar dark region in the agar-gel phantom (Figure 2a) indicates the location of the fiber optic temperature sensor. The phase drift correction to the accumulative MR temperature imaging was validated by comparison with the temperature measurements obtained using the optic temperature sensor. The mean value within 5.25 mm^2^ (7 × 7 pixels in yellow box in Figure 2a) of each accumulative PRFS temperature image was taken, except in the region around the susceptibility artifact (red box in Figure 2a). The measured temperature values (red line in Figure 2b, after phase drift correction) were compared with the fiber optic temperature readings (blue line in Figure 2b, reading at the red box in Figure 2a). The mean phase values in a 5 × 5 pixel area at each of the five oil phantoms (five points in total) were taken to estimate the spatial and temporal variations in the phase drift at each phase change. The dashed box in Figure 2c indicates the area of the phase drift correction, which was interpolated from the five reference points. The estimated phase drift was then subtracted from the original phase change as expressed in Equation (1). Figure 2c,d show the PRFS images before and after the phase drift correction, respectively.

### 2.2. In Vivo Brain Temperature Imaging under General Anesthesia

We scanned the accumulative MR temperature of eight canines (beagle dogs) with a mean weight of 8.9 ± 2.1 kg with various body conditioning scores (BCS, body conditioning score; 1–9, with the higher number indicating greater obesity) that indicate the degree of obesity of individual subjects [14]. Each canine was administered 6 mg/kg of intravenous (IV) propofol (Provive injection 1%, Baxter, Newbury, UK) and scanned in the sternal recumbency position. The canines were mechanically ventilated during the experiments using an animal ventilator (Vetia animal model/ANY VENT, J&TEC, Gimpo, Korea) with a respiration rate of 10 time/minute, and 2–3% isoflurane (Terrell, Piramal Critical Care, Bethlehem, PA, USA) and oxygen (1.0 L/min) were administered. To ensure the safety of the canines during the general anesthesia, the end-tidal carbon dioxide (EtCO_2_), blood pressure, and heart rate were measured using a noninvasive animal patient monitoring system (M20 VET, Mediana, Wonju, Korea). The preparation time of the general anesthesia was shortened as much as possible (approximately 15 min) to minimize the temperature change during the time between the propofol injection to the first MRI phase scan. In accordance with the general practice of veterinary medicine, most of the canines were scanned while covered with a blanket. Two of the eight subjects were scanned without the covering blanket to assess its influence on thermoregulation. Rectal temperatures were continuously measured throughout the period of general anesthesia through the insertion of the fiber optic temperature sensor into the rectum. The experiment lasted for 150 ± 11 min (mean ± standard deviation), depending on the status of the canines; however, if the rectal temperature decreased below 34 °C, the experiment was immediately halted to prevent arrhythmias or other complications and ensure the safety of the canines. The in vivo MR temperature scan protocol and experimental configurations (i.e., MRI system and oil phantom locations) were identical to those used in the agar-gel phantom study. The in vivo brain images of anatomical brain structures including the corpus callosum, lateral ventricles, and the hypothalamus along the axial plane were acquired at every 3-min interval after inducing general anesthesia. After processing all the brain temperature images, we defined specific 5 × 5 pixel regions showing gray and white matter including the corpus callosum and cerebral cortex. Through these 5×5 pixel regions, we acquired the mean and standard deviation temperature values of white and gray matter for comparison and statistical analysis. We also identified the relationship between BCS and core temperature change from the results of the six experiments in which the canines were covered with a blanket. Even though the rate of the temperature change correlates with equilibrium core temperature, the other experiments were halted before the core temperature reached equilibrium for the subject’s safety. Accordingly, the rate of the core temperature change of each of the 6 subjects was obtained by dividing the falling temperature for 2 h from the start of the MRI scans by the number of scans measured during the 2 h period. Each temperature change rate was plotted according to their BCS and fitted to a 1st degree polynomial to find a tendency and correlation (Figure 8).

## 3. Results

### 3.1. Phantom Study

The axial magnitude MR image of the agar-gel phantom is shown in Figure 2a, including the location of five oil phantoms (blue arrows). The accumulative PRFS temperature ($\Delta T_{acc}$) changes are in good agreement with the temperature sensor readings (Figure 2b). The mean difference between the PRFS_acc_ and temperature sensor measurements was approximately 4.3% (Figure 2b red and blue line). Significant differences between $\Delta T_{acc}$ and the temperature sensor measurements were observed before the phase drift correction (black line in Figure 2b).

### 3.2. In Vivo Brain Temperature Imaging

**Figure 3:**
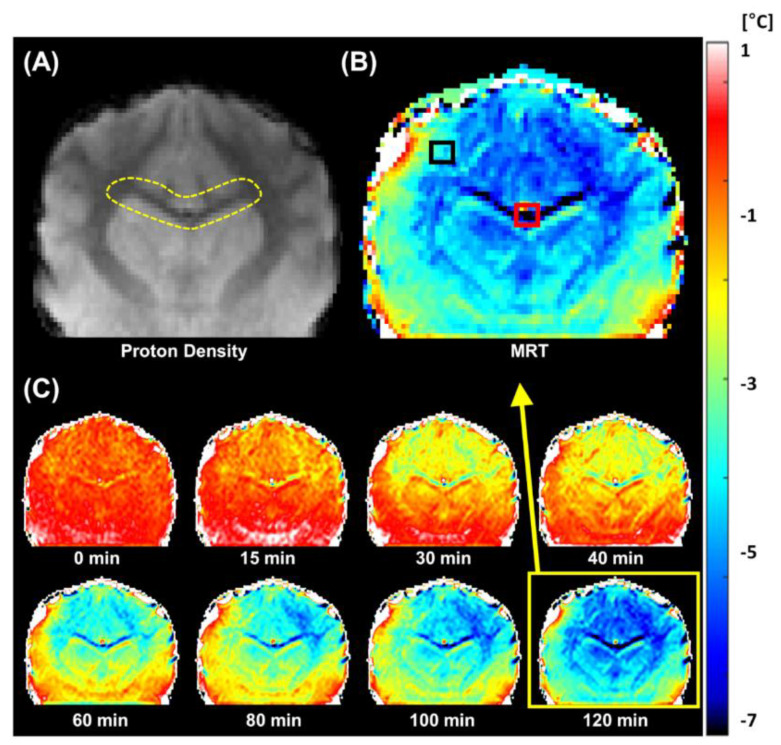
In vivo MR temperature images of canines under general anesthesia. (**A**) Axial MR magnitude image (yellow dashed region: corpus callosum). (**B**) MR temperature image. Corpus callosum (red box, contains white matter only) and cerebral cortex regions (black box, gray matter) of MR temperature image are indicated for temporal temperature changes in Figure 4. (**C**) Selected temporal MR temperature images during general anesthesia.

**Figure 4:**
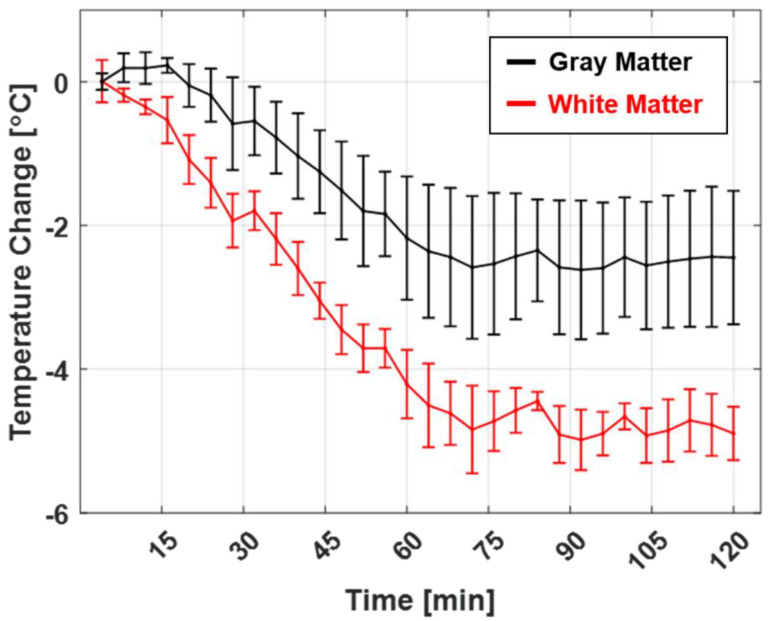
Temperature changes in white and gray matters of six canines under general anesthesia. Mean temperature changes in white matter (corpus callosum, red line, 4.8 ± 0.5 °C) and gray matter (cerebral cortex, blue line, 2.6 ± 0.9 °C) of 6 canines. The error bars indicate the standard deviation of 6 subjects.

**Figure 5:**
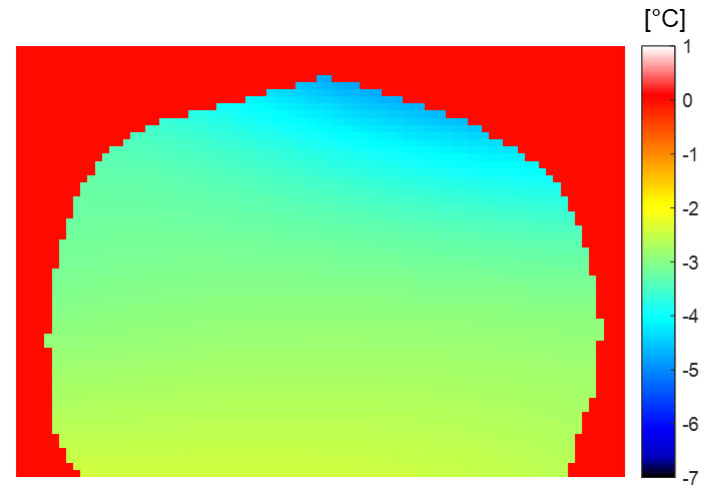
Phase drift map for Figure 3B,C, which is interpolated from the five-point phase measurements using the cylindrical oil phantoms around the canine.

Accumulative in vivo brain MR temperature images were obtained under the same experimental conditions and scan protocol as in the agar-gel phantom study. Figure 3 illustrates the corresponding temperature images. The overall brain temperature was clearly and gradually reduced during the general anesthesia (Figure 3C). The temperature contrast was also observed in different brain tissues. White matter regions, including the corpus callosum (red box in Figure 3B, 5 × 5 pixels), showed more temperature changes than gray matter regions like the cerebral cortex (black box in Figure 3B, 5 × 5 pixels). In the top left in Figure 3C, the contrast in the temperatures of different brain tissues is not particularly distinguishable. As the experiment progressed, the temperature contrast between different brain tissues increased, and the overall brain temperature decreased. The mean and standard deviation of the white matter (corpus callosum) and gray matter (cerebral cortex) in six canines were temporally analyzed (Figure 4). More temperature reductions were observed in white matter (4.8 ± 0.5 °C) than in gray matter (2.6 ± 0.9 °C) regions. In Figure 5, the phase drift map of a canine was shown, which was interpolated from five-point measurements using cylindrical oil phantoms around the canine.

**Figure 6:**
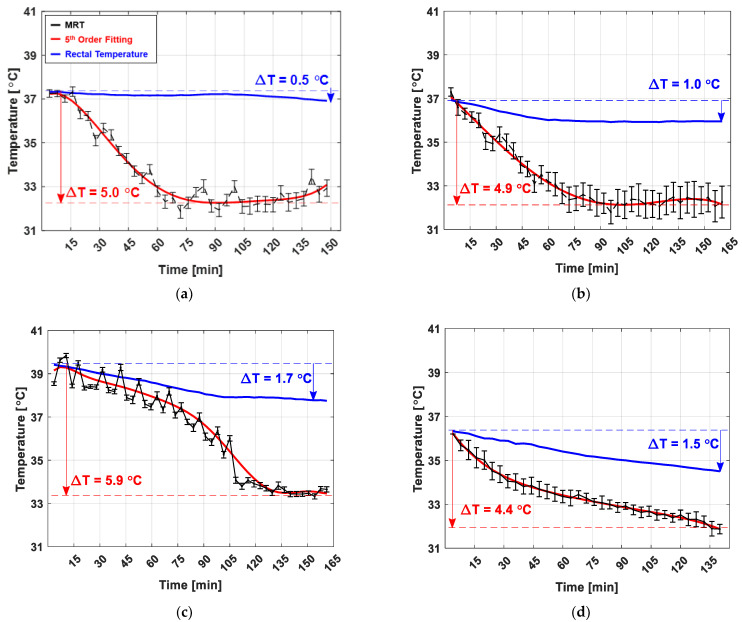

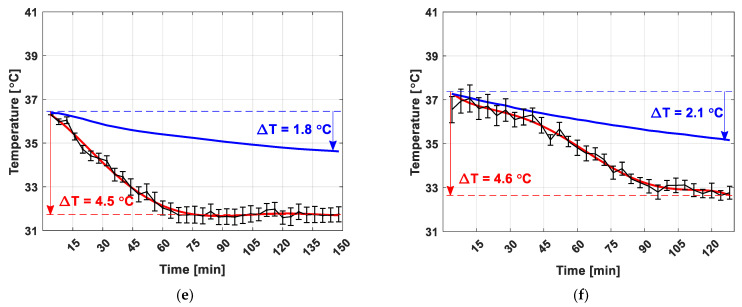
Temperature changes in the white and gray matters of six canines under general anesthesia. Mean temperature changes in white matter (corpus callosum, red line, 4.8 ± 0.5 °C) and gray matter (cerebral cortex, blue line, 2.6 ± 0.9 °C) of six canines under general anesthesia. The error bars indicate the standard deviation in the temperature changes of the six subjects. (**a**,**b**) depict the results for overweight canines (BCS 8); (**c**–**e**) depict those for canines of normal weight (BCS 4–5); and (**f**) depicts the result of underweight canines (BCS 2).

For quantitative analysis, the mean values of the corpus callosum (red box in Figure 3B) were obtained sequentially from each subject; the temperature changes observed in an individual canine under general anesthesia are shown in Figure 6. Owing to the in vivo environment, where breathing is involved, fluctuations of approximately 1 °C were observed in each MR temperature measurement. The error bars in Figure 6 represent the standard deviation of the temperature measurements in the corpus callosum of each canine. To observe the tendency of the temperature variations, corresponding MR temperature data were fitted to a 5th degree polynomial (red line in Figure 6). The starting brain temperature was adjusted to match the rectal temperature (blue line in Figure 6) to facilitate the comparison of the two temperature measurements. The mean and standard deviation of brain temperature reduction of the six canines was 4.8 ± 0.5 °C (white matter), whereas the core body temperature measured rectally was 1.4 ± 0.5 °C. The BCS of each subject had a significant influence on the changes in the core body temperature. The canines whose data is shown in Figure 6a,b were overweight (BCS 8); consequently, the reduction in their core body temperature was relatively low. However, the underweight canine in Figure 6f (BCS 2) underwent a relatively large change in its core body temperature.

**Figure 7:**
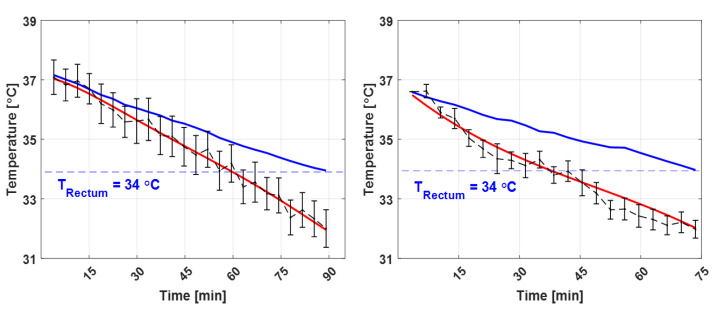
Brain (black dashed line–MR temperature, red line–fitted) and core body (blue line) temperatures under general anesthesia without a blanket. Both canines had normal weights (BCS 4). The experiments were halted because the core body temperature fell below 34 °C.

The results from the two canines that were scanned without the blanket covering (unlike the general practice in veterinary medicine) are shown in Figure 7. The blankets acted like thermal insulation; accordingly, both the brain and core body temperatures abruptly decreased when the blanket was not used. Furthermore, the MR temperature experiments were stopped after a shorter time as the core body temperatures of the canines quickly decreased below 34 °C, owing to the absence of the blanket. Consequently, the brain temperatures of these canines continuously decreased throughout the period of general anesthesia.

## 4. Discussion

One of the particularly interesting results of this study is the variation in the temperature of the different brain tissues, as indicated by the axial MR temperature images in Figure 3 and the statistical analysis in Figure 4. The temperature reduction of white matter was greater than that of gray matter; this was attributed to the thermoregulation mechanism of the brain. The heat transfers via the blood flow and the self-heating mechanism of the brain’s metabolism were the most significant factors affecting the brain temperature [7,15]. Blood serves as the heat transfer medium during thermoregulation. When the metabolism is downregulated due to the effects of general anesthesia, the blood vessels expand and the blood flow increases, significantly affecting the observed heat transfer [16,17]. Furthermore, the neuronal tissues of the central nervous system (CNS) are aggregated in gray matter that comprises numerous neuronal cell bodies and neuropils and constitutes the primary metabolic site. This region includes several blood vessels that supply the oxygen required for metabolism. In contrast, white matter comprises myelinated axons in bundles of nerve fibers that enable signal transfers in the CNS [18,19]. Owing to these differences in composition and role, gray matter contains more blood and water in the cell tissues [20]. Thus, the reduction in the temperature of gray matter is less than that of white matter because the additional blood vessels and metabolism of the former contribute toward thermoregulation [7,15].

Previous invasive temperature monitoring techniques could not accurately demonstrate the reduction in temperature caused by only the effects of general anesthesia because of the additional temperature changes due to the inflammation around the direct thermometer insertion into the brain [7]. However, the accumulative PRFS can noninvasively detect the intermediate process contributing to brain temperature reduction under general anesthesia. Figure 6 presents the temporal MR temperature data acquired from the corpus callosum (red box in Figure 3B). The reduction in the core body temperature under general anesthesia was attributed to the increased threshold for the activation of thermoregulation, the reduction in metabolism, and the increased heat loss due to vasodilatation [4,21]. The brain has a more complex thermoregulation mechanism than other parts of the body owing to the metabolic reactions [22,23,24]. The brain has a higher temperature than the rest of the body due to the heat generated via metabolism and regulates its temperature by releasing heat via blood circulation [7,25]. However, under the effects of general anesthesia, the metabolism of the brain is significantly downregulated; the temperature is also reduced accordingly [26,27]. In other words, the significant and rapid reduction in brain temperature is attributed to the downregulated metabolism and the heat lost to the ambient environment. In contrast, the core body temperature decreases to a lesser extent because heat is primarily lost to the ambient environment [15].

**Figure 8:**
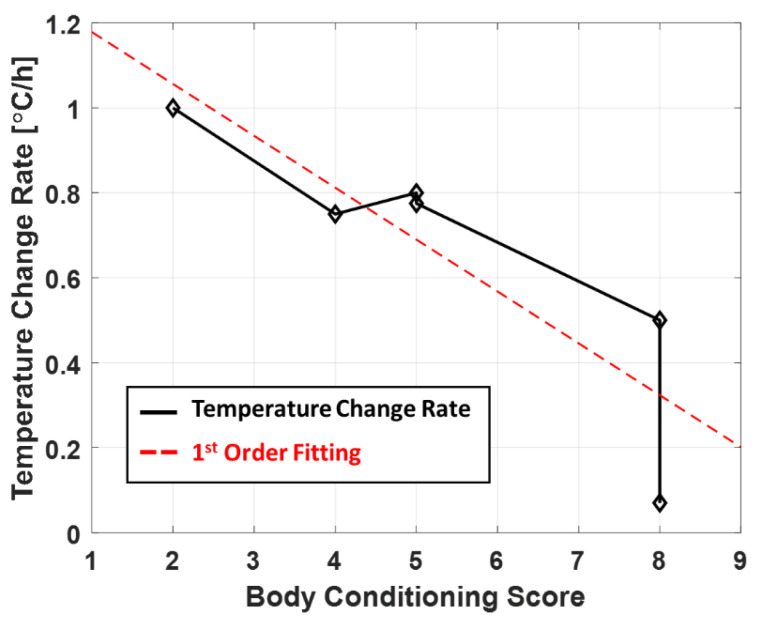
Correlation between body conditioning score (BCS) and core (rectum) temperature change rate. The subjects with a lower BCS, i.e., underweight subjects, showed the highest rate of temperature reduction (°C/h) during general anesthesia. The correlation coefficient between BCS and the rate of core temperature change is −0.88 (*p* = 0.0046, *α* < 0.05).

The size, weight, and BCS of the canines were the primary factors that significantly affected the variations in their core body temperatures. Canines with a higher BCS underwent a smaller reduction in core body temperature (Figure 6). The relationship between the core body temperature changes and the BCS is shown in Figure 8. The correlation coefficient was −0.88 (*p* = 0.0046, *α* < 0.05), indicating that subjects with a lower BCS showed greater temperature reductions under general anesthesia. In general, large canines have a low risk of hypothermia when under general anesthesia because they have a low ratio of body surface and skin [28,29]. Moreover, if the canine is overweight, the increased fat deposition improves the heat insulation of the core body [30,31]. In particular, the core body temperature of the most overweight canine (BCS 8), which was also covered with a blanket (Figure 6a), remained almost constant throughout the MR temperature experiments, whereas its brain temperature decreased considerably. This insulation effect, afforded by fat, is similar to that provided by the blanket commonly used in veterinary medicine when administering general anesthesia. The insulating effect of the blanket can be determined by comparison with the temperature variations of the canines that were not covered with blankets (Figure 7). The canines that were not covered with the blanket underwent a steeper temperature change than those covered with blankets. Although the experiments on the canines without blankets were halted, it could be expected that if the experiment was continued, a temperature plateau below 34 °C would be attained [28,29]. Accordingly, it can be inferred that the plateau of the core body temperature is dependent on the degree of insulation, which happens to be provided by body fat and the use of a blanket. In contrast, the brain temperature in all experiments decreased more rapidly than the core body temperature, regardless of the BCS or the use of blankets. Thus, it is evident that the brain temperature can vary significantly from the core body temperature, which may lead to an underestimation of the brain temperature by commonly used rectal temperature measurements. Therefore, it is essential to accurately monitor the brain temperature when administering general anesthesia to avoid complications such as CNS fatigue, metabolic downregulation, variations in insulin resistance, and autophagic cell death that are caused by brain hypothermia [32,33,34].

Fluctuations in the MR temperature data were observed during the in vivo experiments, wherein forced breathing was performed after the administration of general anesthesia using a ventilator by 10 times per minute, which is shorter than the phase scanning time (43 s). This breathing can increase the inhomogeneity of the magnetic field (*B*_0_), thereby potentially altering the MRI phase signals, which, in turn, alter the temperature images. Similarly, the vascular pulsation can also increase the *B*_0_ inhomogeneity as well. Since the extent of this effect can vary depending on the time between imaging and the breathing cycle [35,36], the degree of fluctuation varied between experiments and subjects. Nevertheless, these fluctuations were negligible when compared with the temperature reduction induced by the effects of general anesthesia. Thus, the tendency of temperature change was examined by fitting the data to 5th order polynomials.

A more careful PRFS experimental design needs to be followed for successful in vivo temperature monitoring. Since the PRFS technique is insensitive to the adipose tissue, the temperature changes within the adipose tissues cannot be technically monitored. If the targeting biological tissues are fatty tissue, other in vivo MRT techniques should be considered such as T_1_, T_2_, or diffusion-based MRT [9]. They utilize changes in time constants due to the temperature changes, but they do suffer from a lower signal-to-noise ratio [8]. Another technical point to consider is the local/global temperature increase of a subject during the temperature monitoring. Applying RF pulses to a subject, for MR signal generation, is fundamentally needed. However, if the RFs are intensively applied to a subject, RF pulsing itself may cause an unwanted local/global temperature increase of a subject. Setting a longer repetition time (TR), lower flip angle (FA), or smaller number of imaging slices are the possible remedies for lowering the unwanted temperature increase.

## 5. Conclusions

In this study, the noninvasively measured brain temperature of canines was compared with their core body temperature under general anesthesia. The quantitative temperature images showed that under general anesthesia, the core body temperature decreased by 1.4 ± 0.5 °C, whereas the brain temperature (white matter) decreased by 4.8 ± 0.5 °C due to metabolic downregulation. In particular, the reduction in brain temperature differed depending on the type of brain tissue; the temperature reduction of white matter was greater than that of gray matter. Furthermore, the temperature difference in different brain tissues were interpreted based on the thermoregulation mechanism of the brain, which is more complex than that of the body’s core. For this purpose, the accumulative form of the PRFS method was used. Future studies will aim to combine more quantitative MRI measurements with current MR thermometry to improve our fundamental understanding of the thermoregulation mechanism of the brain.

## Data Availability

The data presented in this study are available on request from the corresponding author.
